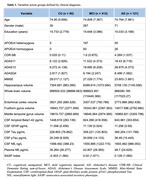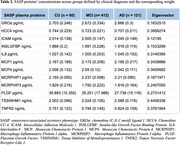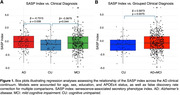# Senescence‐associated secretory phenotype (SASP) index in individuals across the Alzheimer’s disease continuum

**DOI:** 10.1002/alz.092727

**Published:** 2025-01-09

**Authors:** Alvaro de Oliveira Franco, Thomas Hugentobler Schlickmann, João Pedro Ferrari‐Souza, Marco Antônio de Bastiani, Raphael Machado Castilhos, Eduardo R. Zimmer

**Affiliations:** ^1^ Hospital de Clinicas de Porto Alegre, Porto Alegre, Rio Grande do Sul Brazil; ^2^ Universidade Federal do Rio Grande do Sul, Porto Alegre, Rio Grande do Sul Brazil; ^3^ Hospital de Clínicas de Porto Alegre, Porto Alegre, Rio Grande do Sul Brazil

## Abstract

**Background:**

Cellular senescence (CS) is a hallmark of aging. Senescence‐associated secretory phenotype (SASP) index was recently developed based on the levels of blood SASP‐related proteins. Although aging is a primary risk factor for Alzheimer's disease (AD), the role of CS in this neurodegenerative condition is not well understood. Here, we investigated the association of the SASP index (SI) with classical markers of AD.

**Methods:**

We assessed 593 participants (60 cognitively unimpaired [CU], 412 with mild cognitive impairment [MCI], and 121 with AD dementia), from the Alzheimer's Disease Neuroimaging Initiative (ADNI) cohort (Table 1). SI was derived through a weighted sum of the standardized concentrations of 12 available SASP proteins, each weighted according to the eigenvectors from a Principal Component Analysis of these proteins (Table 2). Associations were tested using generalized linear models adjusted for age, sex, education, and *APOE*ε4 status. Statistical analyses were performed in the R environment, significance set at p < 0.05, and multiple comparisons correction was performed using the false discovery rate.

**Results:**

Regression analysis demonstrated that CU individuals showed lower SI values than MCI (p = 0.044) and AD dementia (p = 0.039) individuals. We found that the SI was positively associated with age (p < 0.001), cerebrospinal fluid (CSF) neurofilament light (NfL) (β = 94.838, p = 0.007), plasma NfL (β = 4.08, p‐value < 0.001) and Clinical Dementia Rating sum of the boxes (β = 0.12, p = 0.004). On the other hand, the SI was negatively associated with the middle temporal gyrus volume (β = ‐208.81, p = 0.013), with a negative trend with hippocampus volume (β = ‐56.15, p = 0.053). Additionally, the SI showed no significant correlation with CSF Aβ42, CSF t‐tau and p‐tau181, CSF GFAP, education level, and the whole brain, entorhinal gyrus, fusiform, and entorhinal cortices volumes.

**Conclusions:**

Here, we showed that the SI closely associates with hallmark features of AD, such as age, cognition, fluid, and imaging biomarkers. Our results help to better understand the role of aging in AD and suggest that the SI can be used as a proxy for CS in AD.